# Determination of vancomycin minimum inhibitory concentration for ceftazidime resistant *Streptococcus pneumoniae* in Iran

**DOI:** 10.1186/s12941-014-0053-1

**Published:** 2014-11-11

**Authors:** Ramezan Ali Ataee, Samira Habibian, Ali Mehrabi-Tavana, Zyanab Ahmadi, Nematollah Jonaidi, Mahmood Salesi

**Affiliations:** Health Management Research Center, Baqiyatallah University of Medical Sciences, Tehran, Iran; Molecular Biology Research Center, Baqiyatallah University of Medical Sciences, Tehran, Iran; Department of Infection Diseases and Health Research Center, Baqiyatallah University of Medical Sciences, Tehran, Iran; Department of Statistic and Health Research Center, Baqiyatallah University of Medical Sciences, Tehran, Iran

**Keywords:** Ceftazimdime resistance, Etest, *Streptococcus pneumoniae*, Vancomycin, Minimum inhibitory concentration, Maximum zone of inhibition

## Abstract

**Background:**

In the context of growing health concerns over antibiotic resistance, the evaluation of the minimum inhibitory concentration (MIC) of vancomycin for *Streptococcus pneumoniae* (*S. pneumoniae*) strains resistant to ceftazidime becomes important for guiding health policy makers. The aim of this study was to determine vancomycin MIC of ceftazidime resistant *S. pneumoniae* strains.

**Methods:**

Fifty identified serotypes of ceftazidime resistant *S. pneumoniae* strains were included in the study. The vancomycin MIC of the above mentioned bacteria was determined based on the 0.5 McFarland standards, by using a microdilution broth and the Etest method.

**Results:**

The results showed that out of 50 ceftazidime resistant strains of *S. pneumoniae,* 46 strains (92%) have shown a vancomycin MIC ≤0.19 − 0.1.5 μ*g*/ml and only four strains (8%) have shown a vancomycin MIC equal to 1.5 μ*g*/ml and the related maximum zone of inhibition was of 10 millimeter diameters.

**Conclusions:**

The results of this investigation point out the emergence of *S. pneumoniae* strains with a vancomycin MIC ≥1.5 μ*g*/ml, which were resistant to ceftazidime. This finding uncovers a major health concern: a vancomycin MIC higher than 1.5 μ*g*/ml and maximum zone of inhibition of only 10 millimeter. These findings represent an important warning for health authorities globally, concerning the treatment of patients, as the occurrence of *S. pneumoniae* strains with decreased vancomycin susceptibility has been demonstrated.

## Background

*Streptococcus pneumoniae (S. pneumoniae)* is one of the major causes of invasive bacterial disease, such as pneumonia and meningitis, leading to increased morbidity and mortality rates, especially among the elderly and infants [1]. In addition, the results of a recent study have showed that *S. pneumoniae* is colonizing the nasopharynx and, on a single occasion, appropriate culturing yields pneumococci in 5 − 10% of healthy adults and 20 − 40% of healthy children. With regard to healthy carriers, children are at a higher mortality risk compared to elderly and also, have an increased risk of pneumococcal infection. It has been shown that by repeated attempts and with the improvement of bacteriological methods, the percentage of nasopharyngeal carriers rises from 40% to 60%, or greater, in all age groups [2]. This finding may be an explanation of the development of antibiotic-resistant strains. Because penicillin is the first drug of choice in the treatment of pneumococcal infections, yet the incidence of penicillin resistance in *S. pneumoniae* has increased dramatically, this phenomenon becomes a major health concern worldwide [3,4]. Results of a previous study have revealed that penicillin resistance rate was 65% in children, versus 22% in adults [5]. Furthermore, the increase of multidrug resistance in *S. pneumoniae* isolates makes the treatment of these infections even more difficult [6]. However, vancomycin is the last drug of choice in the case of pneumococcal meningitis caused by non- susceptible to penicillin strains and it has been used more frequently for severe, invasive infectious disease, where resistance to other antibiotics was present. This translates into the fact that vancomycin plays a key role in the management of multidrug-resistant pneumococcal infections [7]. These data emphasized antibiotic susceptibility surveillance systems are an important tool for preventing the emergence and spread of multidrug-resistant pathogens. Only limited data available suggest that imipenem and vancomycin may be the most active against penicillin-resistant strains of *S. pneumoniae* [8,9]. However, the results of another study, in Serbia, indicated that multidrug resistance was found in one third of pneumococcal isolates, whereas all isolated pneumococcal strains were susceptible to vancomycin, linezolid, fluoroquinolones, telithromycin and rifampicin [1]. Nevertheless, vancomycin tolerant *S. pneumoniae* strains have been reported and characterized [10]. In order to determine vancomycin tolerance (VTSP) or vancomycin non susceptible of *S. pneumoniae*, continuous and comprehensive monitoring of minimum inhibitory concentration (MIC) may be successfully associated with the management and treatment [11]. Recently, based on research results, the ability to survive exposure to an antibiotic, through the loss of autolysin activity or triggering, is termed “tolerance” [12,13]. As a result, Normark BH and his colleagues showed the limiting criteria for pneumococcal vancomycin tolerance. They showed that the mean log kill value was three (SD ± 0.3) and the mean loss of optical density (OD) was 89% (SD ± 3). The results of these investigators’ studies indicate that, for the pneumococcal lyt 4–4 strain, the log kill was 1.4 (SD ± 0.3) and the loss of OD was 19% (SD ± 12). Based on these limitations, they have defined vancomycin tolerance as a log kill of ≤2 and as OD loss of ≤43% [14]. As can be seen, the interpretation of data on vancomycin tolerance is very difficult and may be impossible for clinical laboratory technicians. However, recent data show that the MIC of the antibiotic has changed and it has been suggested that this tolerance could result in the development of drug resistance [15]. The initial therapy for *S. pneumoniae* infections based on accurate antibiotic resistance patterns among isolates in every region becomes a necessity and leads to a minimization of the development of antibiotic resistance [16]. The results of a previous study of our team revealed that 48% of *S. pneumoniae* strains were resistant to ceftazidime [17]. Therefore, specific and reliable studies are needed to detect and evaluate the vancomycin MIC among ceftazidime resistant pneumococcal clinical isolates in Iran. Therefore, the aim of this study was to determine vancomycin MICs, with the distribution of ceftazidime resistant *S. pneumoniae* serotypes in Iranian patients, by using serial microdilution and the Etest method.

## Materials and Methods

In this investigation, vancomycin MIC strips (MIC Test Strip, Liofilchem, Roseto degli Abruzzi Italy) were used. Serial microdilutions of native manufactured vancomycin (500 mg/vial, Dana Pharmacological Co, Tabriz, Iran) were performed, while Mueller-Hinton broth (Merck, Darmstadt, Germany), defibrinated lysed sheep blood (Baharafshan Co., Teheran, Iran), 96-well microplate (Technogen Spa, Pontenure Piacenza, Italy) and injectable distilled water were used.

### Bacterial strains

A total of 50 clinical *S. pneumoniae* strains, with previously determined serotypes [1] and also resistant to ceftazidime, were subjected to vancomycin MIC determination. By using standard procedures recommended, lyophilized strains were recovered. In this investigation, antimicrobial susceptibility testing was performed according to Clinical and Laboratory Standards Institute (CLSI) guidelines [18]. The antimicrobial susceptibility patterns of the strains to vancomycin were determined by using both microdilution and the Etest method as confirmatory testing.

### Inoculums size

Bacterial suspensions for inocula were prepared from 18 hours BHI broth medium equivalent to McFarland 0.5 standard, which provides turbidity comparable with that of a bacterial suspension containing 1.5 × 10^8^ colony-forming units (CFU)/ml. In fact, a McFarland 0.5 standard corresponds to an OD 600 between 0.08 and 0.1.

### Vancomycin minimum inhibitory concentration determination by etest method

Each of the bacterial strains were separately inoculated on Muller-Hinton Agar plate (MHAP) containing 5% defibrinated lysed sheep blood and a vancomycin MIC test strip (Liofilchem, Roseto degli Abruzzi, Italy) was placed on the inoculums lawn. Briefly, the procedure was performed as follows: a sterile swab was sunk into the bacterial suspension and then inocula onto Muller-Hinton 5% defibrinated sheep blood agar plates and after 15 to 20 min, when the bacterial suspension were absorbed onto the medium, vancomycin MIC strips were applied on the plate. The plates were incubated at 37°C for 24 h in an atmosphere containing 3− 5% CO_2_.

After the incubation period, the elliptical zone of inhibition was measured and the MIC of each strain was determined, separately. The MIC was the point where the elliptical zone of growth inhibition intersected the MIC scale on the vancomycin strip. The antibiotic gradient scale on the vancomycin MIC strip was 0.016 − 256 μg/ml.

### Vancomycin minimum inhibitory concentration determination by serial microdilutions method

Based on our previous reports and references of bacterial strains applied [19], the stock solution of vancomycin was prepared. Serial concentrations with twice the anterior value progressive rate (0.5, 1, 2, 4, 8, 16, 32, 64, 128 μg/well) of vancomycin were then prepared and loaded into defined rows of 96 well microplates from the antibiotic stock solution. Plates were incubated at 35°C for 24 hours, until evaporation and finalization of the process of loading the antibiotic into the plate. The plates were stored in a refrigerator at 4°C for 3 hours.

The designing of the 96-well microplate (for preparation of the serial dilutions of vancomycin) was performed as follows: one row for negative control, one row for positive control and three rows for tests were marked, respectively. Therefore, each 96-well microplate was considered to measure the antibiotic sensitivity of two bacterial strains. The negative control rows for each case consisted of the serial dilutions of vancomycin (0.5, 1, 2, 4, 8, 16, 32, 64, 128 μg/well), culture media (Mueller-Hinton broth containing 3% defibrinated sheep blood) and no bacterial suspension. The positive control rows consisted of culture media (Mueller-Hinton broth containing 3% defibrinated sheep blood) and the bacterial suspension. The positive control rows were lacking vancomycin. The three rows of microplates for tests with equal conditions were used for each bacterial strain testing. The test rows were composed of serial dilutions of vancomycin (0.5, 1, 2, 4, 8, 16, 32, 64, 128 μg/wells, respectively), culture media and bacterial suspension. After inoculation and incubation, the plates were subjected to scanning at 450 nm of wavelength ELISA reader. Data were analyzed using unilateral analysis of variance (ANOVA). In our statistical analyses, α =0.05 was considered acceptable for significant variation and the results were analyzed using SPSS Version 16.0 Software (SPSS Inc., Chicago, IL, The USA).

### Molecular assay for resistance gene

In this study, we used previously published collections of primer pairs [20] or designed them based on the nucleotide reference GeneBank. Polymerase chain reactions (PCR) for *PBP2b*, *PBP2x*, *gyrA*, *vex2* and *vncS* genes, which amplified 1500, 1920, 2100, 400 and 1000 bp fragments were performed respectively (Table 1).

**Table 1:** **The sequences of the primer pairs used in this study**

**Gene**	**Nucleotide sequences**	**Charactristics**		
		**Size of amplified product (bp)**	**Induce resistant to**	**Reference**
***PBP2b***	F – 5′ GAT CCT CTA AAT GAT TCT CAG GTG G 3′	1500 bp	Penicillin	[20]
	R – 5′ TGG TGT TCG TGT GGC TCC TC 3			
***PBP2x***	F- 5′ GAT TGC TGA GGA TGC AAC CTC TTA TAA TGT CTA TG 3′	1920 bp	Ceftazidime	
	R −5′ GCC TTG AAA TTC AAG TTC TAT ATT GAG CCA CTT AGC 3′		Ceftriaxon	
***gyrA***	F – 5′ CCG TTG TGA AAG TCA CTA TCT G 3′	2100 bp	Ciprofloxacin	In this study
	R – 5′ AGT TGC TCC ATT AAC CA 3′			
***vncS***	F – 5′ AAA CAA TTG ACC TCC TCC AGA TG 3′	1000 bp	Vancomycin	
	R – 5′ TTC TAA CTC CAT CTA TGT AAA CC 3′			

In our study, the mentioned primer pairs were selected and synthesized by CinnaGen Co, Teheran, Iran. For the detection and amplification of the above mentioned genes, overnight grown bacterial cells of the late exponential phase were subjected to DNA extraction, using the salting out method [21]. The 25 μL volume PCR reaction master mixes were prepared in 0.2 mL microcentrifuge tubes. An individual reaction was carried out as follows: 2.5 μL PCR buffer (10x), MgCl_2_ (50–150 mM gradient), 0.5 μL dNTPs (10 mM), 0.5 μL of each primer (100 pm/μl), 0.5 μL *Taq* DNA polymerase (5 unit/μl), 1.5 μL template DNA and 18.5 μL sterile deionized water. All the materials used in PCR reactions were purchased from CinnaGen Co., Teheran, Iran. Thermal cycling was performed in the Analytic Jena PCR system (Analytic Jena AG, Jena, Germany). The PCR process included denaturalization at 94°C for 6 min, followed by 35 amplification cycles of 94°C for 45 s, 61°C for 30 s, 72°C for 1 min and with 5 min final extension at 72°C. For the analysis of the PCR products, 1.5% agarose gel containing 1X TBE buffer was prepared and was run at 100 V for 40 min. For determining the size of the PCR products, we have used a 50 bp DNA ladder.

## Results

Demographic analysis revealed that the mean age of patients participating in this study was 52 years old (16 to 73 years old), of which 30 (60%) were women and 20 (40%) were men.

### The results of vancomycin susceptibility pattern

**Figure 1:**
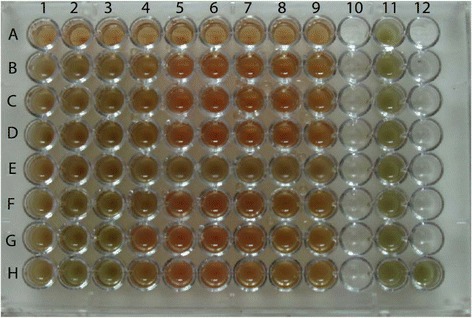
**Illustration an example of the 96 well micro plate’s broth microdilution method for two** ***S. pneumoniae*** **strains vancomycin assay.** In this plate the 9 wells in **A** row is negative control and two three test rows (rows **B**, **C** and **D** for strain one and the rows **F**, **G** and **H** for strain two) for vancomycin MIC determination which contain the serial concentration of 0.5 to 128 μg/well of vancomycin respectively. Nine wells of **E** row and well on column 11 are marked as positive control.

The results of broth microdilution method with vancomycin gradient (concentration) ranges of 0.5, 1, 2, 4, 8, 16, 32, 64, 128 μg/well revealed that 46 isolates (92%) with MIC ≤1.5 μg/well were obtained. Four strains (8%) with MIC ≥1.5 μg/well, one of them with a MIC =8 μg/well (Figure 1), were detected. The results of vancomycin MIC determined by the Etest and broth microdilution method for these strains were the same. In fact, the comparative results of broth microdilution and Etest method revealed 46 isolates (92%) with a MIC ≤1.5 μg/ml, three strains (6%) with MIC ≥1.5 ≤ 6 μg/ml and one strain (2%) with MIC 8 μg/ml. However, analysis of the Etest results indicated that the lowest MIC, was 0.125 μg/ml, belonged to serotype 4, followed by serotypes 6 and 5, which were isolated from different wounds, while three strains from the lungs, with a MIC =0.25 − 0.75 μg/ml and one strain with a MIC =0.38 μg/ml, isolated from the blood, belonged to serotype 1 (Table 2).

**Table 2:** **Results of** ***S. pneumoniae*** **serotype distribution and vancomycin MIC by Etest strip and broth microdilution method, containing vancomycin gradients of 0.016–256** μ**g/ml and 0.5–128** μ**g/well respectively**

**Site of infection**	**Internal code number**	**Serotype or serogroup**	**Minimum inhibitory concentration ranges**				**Maximum zone of inhibition**
			**MIC ≥0.1 ≤ 1** **μg/ml**	**MIC ≥1 ≤ 2** **μg/ml**	**MIC ≥2 ≤ 6** **μg/ml**	**MIC ≥6 ≤ 10** **μg/ml**	
**Wounds**	**104**	**6**	**0.38** **μ** **g/ml**				**2.3 mm**
	**105**	**5**	**0.38** **μg/ml**				**2.1 mm**
	**381**	**10**	**1** **μg/ml**				**1.8 mm**
**Lung**	**201; 102; 13; 120**	**6**	**0.38** **μg/ml**				**2.9 mm**
			**0.50** **μg/ml**				**1.8 mm**
			**0.75** **μg/ml**				**2.2 mm**
			**1** **μg/ml**				**1.7 mm**
	**106; 125;777**	**1**	**0.25** **μg/ml**				**2.3 mm**
			**0.38** **μg/ml**				**2 mm**
			**0.75** **μg/ml**				**2 mm**
	**501**	**4**	**0.125** **μg/ml**				**1 mm**
	**124; 130; 83**	**2**	**0.19** **μg/ml**				**2.1 mm**
			**0.75** **μg/ml**				**1.5 mm**
			**1** **μg/ml**				**2 mm**
	**6; 117**	**7**	**0.5** **μg/ml**				**2 mm**
			**0.75** **μg/ml**				**1.8 mm**
	**34; 107; 48;28**	**8**	**1** **μg/ml**				**2.2 mm**
				**1.5** **μg/ml**			**3 mm**
			**1** **μg/ml**				**2 mm**
			**1** **μg/ml**				**2.4 mm**
	**202**	**G**	**1** **μg/ml**				**2 mm**
**Blood**	**133**	**1**	**0.38** **μg/ml**				**2.1 mm**
	**39**	**2**	**1** **μg/ml**				**2 mm**
	**116**	**4**	**0.5** **μg/ml**				**2.3 mm**
	**131; 121**	**6**	**0.25** **μg/ml**				**2.5 mm**
			**0.38** **μg/ml**				**2.5 mm**
	**89**	**G**	**0.50** **μg/ml**				**2 mm**
	**126; 40**	**17**				**8** μ**g/ml**	**1 mm**
				**1.5** **μg/ml**			**1 mm**
	**35**	**18**	**0.75** **μg/ml**				**2.2 mm**
**Eye**	**101**	**2**	**0.38** **μg/ml**				**2.5 mm**
	**5; 996; 208; 129**	**6**	**0.38** **μg/ml**				**2.5 mm**
			**1** **μg/ml**				**1 mm**
			**1** **μg/ml**				**2 mm**
			**1** **μg/ml**				**1/5 mm**
	**502; 50; 53; 7**	**19**	**0.38** **μg/ml**				**2.1 mm**
			**0.5** **μg/ml**				**2 mm**
			**0.5** **μg/ml**				**2.5 mm**
			**1** **μg/ml**				**2.5 mm**
	**14**	**14**	**0.5** **μg/ml**				**1.7 mm**
**CSF**	**51**	**8**	**1** **μg/ml**				**3 mm**
	**109; 111**	**20**		**1.5** **μg/ml**			**2 mm**
					**6** **μg/ml**		**1 mm**
**Throat**	**128**	**7**	**1** **μg/ml**				**2 mm**
	**123**	**20**			**4** **μg/ml**		**1 mm**
	**103**	**4**	**0.38** **μg/ml**				**2.4 mm**
	**108**	**14**			**5** **μg/ml**		**1 mm**
**Nostrils**	**778; 49**	**7**	**0.5** **μg/ml**				**1.5 mm**
			**0.5** **μg/ml**				**2.5 mm**
**Sinus**	**115**	**20**	**1** **μg/ml**				**1.7 mm**

Figure 1 shows the example of a 24 hours incubation period of a 96-well microplate used for measuring the antibiotic sensitivity of two bacterial strains. The results of the subculturing of each well showed that the vancomycin MIC for *S. pneumoniae* strains was the same as of the Etest strip results, which are displayed in Table 2. The unilateral ANOVA of the OD of each well at time 0 and 24 hours after incubation of the bacterium with different concentrations of antibiotics per well have shown the significant differences (P ≤0.02).

The results of this study revealed that the vancomycin MIC of 92% of pathogenic *S. pneumoniae* is 0.19 − 1.5 μg/ml, as a vancomycin susceptible strain, while 8% of them have a vancomycin MIC ≥1.5 μg/ml, being considered with decreased susceptibility to vancomycin or tolerant, which belonged to serotype 17 and was isolated from the blood.

**Figure 2:**
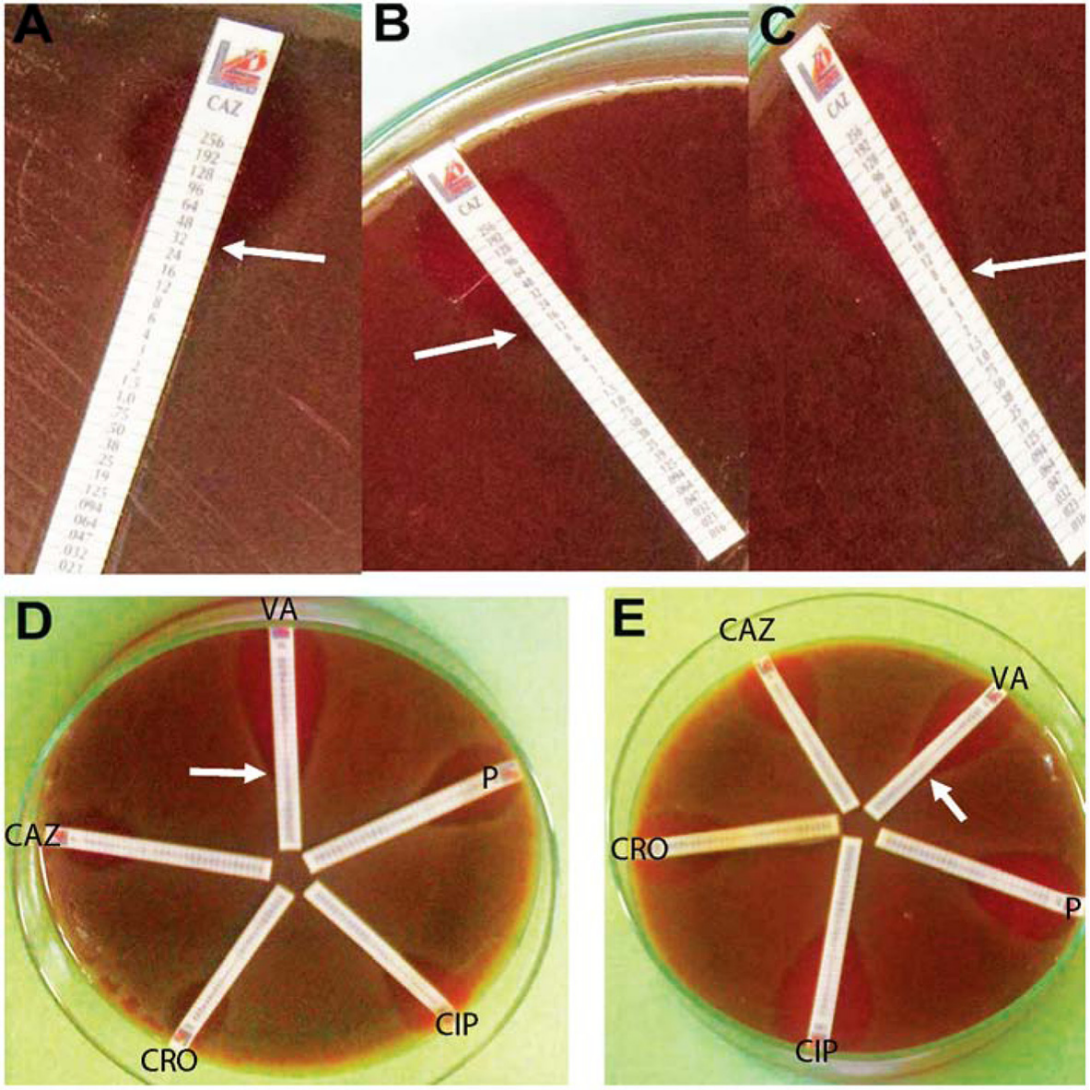
**Sections A − C show the elliptical zone of inhibition of three S** ***. pneumoniae*** **strains around ceftazidime MIC strip, with MIC =32, 16 and 6** **μg/ml of ceftazidime, respectively.** In Part **D** and **E**, antibiotic susceptibility assay plates using Etest strips for penicillin, vancomycin, ceftazidime, ceftriaxone and ciprofloxacin are shown for two *S. pneumoniae* strains. Arrays in part **D** and **E** are shown the vancomycin MIC = 1.5 and 4 μg/ml respectively.

The results of the susceptibility pattern obtained in this study have shown that the most frequent *S. pneumoniae* strain with sensitivity to vancomycin was serotype 6, isolated from lung. In Figure 2, rows A − C showed the three Ceftazidime resistant *S. pneumoniae* strains with MICs of 6, 12 and 32 μg/ml, respectively, while part D and E showed the two S*. pneumoniae* strains with decreased vancomycin sensitivity, with a MIC ≥1.5 μg/ml.

The key finding of this study is shown in the last column of Table 2. By comparing the obtained growth zone of inhibition, we can estimate the vancomycin susceptibility rate or tolerance of the bacterial strains. For example, the vancomycin MIC for *S. pneumoniae* serotype 4 was 0.125 μg/ml, while vancomycin MIC for serotypes 17 and 20 were 8 and 6 μg/ml, respectively, with the same zone of inhibition.

### Results of molecular assay

**Figure 3:**
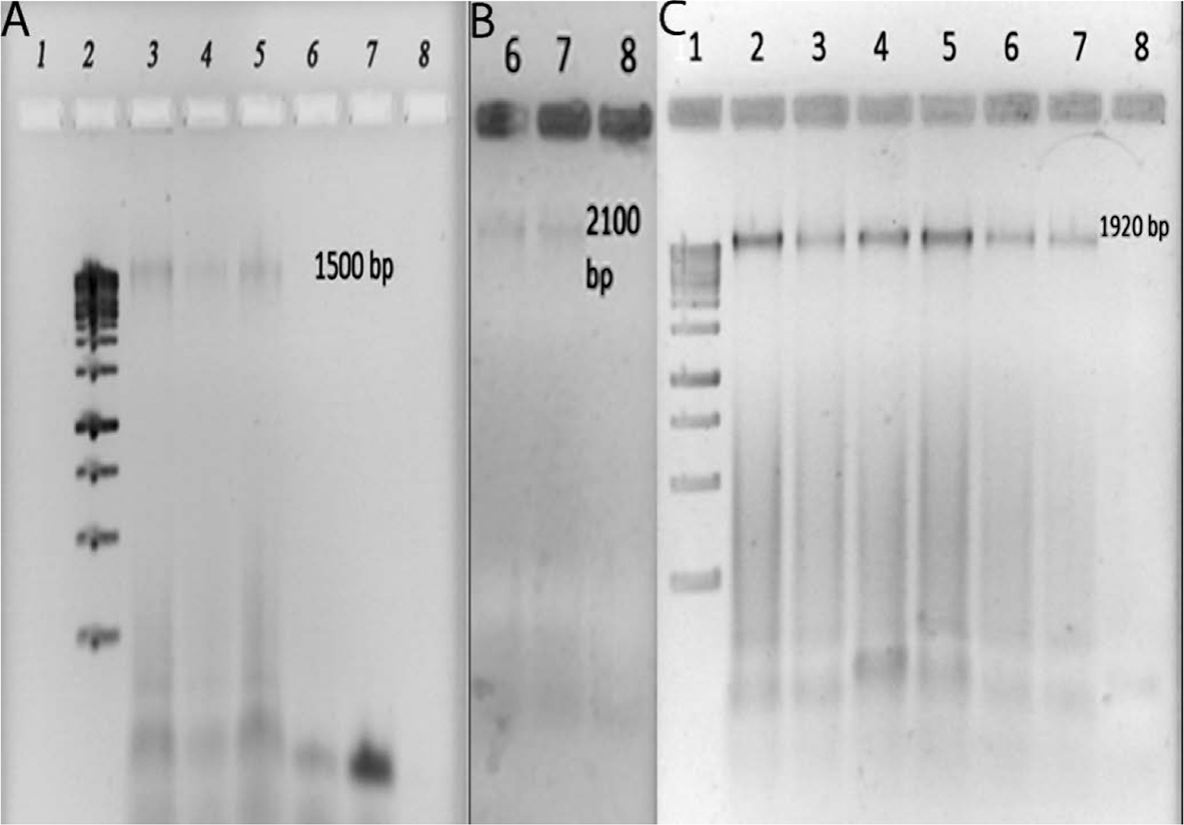
**Sections A, B and C depicts the amplified 1500, 1920** **and 2010bp fragments of** ***PBP2b,*** **PBP2x and** ***gyrA*** **that introduces resistance to penicillin, ceftazidime and or ceftriaxone and ciprofloxacin.**

The results of the molecular assay for *vncS* and *vex2* genes revealed that the used primer pairs in this study were not able to amplify the coding sensor histidine kinase sequences as vancomycin tolerant in all 50 isolated strains of *S. pneumoniae*, while the results of PCR for *PBP2b*, *PBP2x* and *gyrA*, as determinants of penicillin, ceftriaxone, ceftazidime and ciprofloxacin resistance, amplified the fragment of their related genes (Figure 3). On the other hand, the PCR protocol shows no vancomycin tolerant genes in the studied *S. pneumoniae* strains.

The outlined results of this study revealed that the vancomycin MIC of 92% (46 strains) of pathogenic *S. pneumoniae* is 0.19 − 1.5 μg/ml, while 8% (four strains) have a vancomycin MIC ≥1.5 μg/ml. The related genes resistant to penicillin, ceftriaxone, ceftazidime were detected in several *S. pneumoniae* strains, which had been marked as β-lactam resistant isolates.

## Discussion

Pneumococcal resistance to antibiotics, especially to penicillin, poses an increased therapeutic concern [22,23]. In addition, vancomycin tolerant *S. pneumoniae* had been observed in three clinical isolates in the early ’90s [7]. However, given the fact that the antibiotic of choice for treating meningeal infections caused by penicillin resistant *S. pneumonia*e strains is vancomycin and also, empirical therapy for purulent meningitis caused by head trauma or post neurosurgery are an indication for vancomycin plus either ceftazidime, cefepime or meropenem, researchers have recommended the necessity to assay the efficiency of vancomycin in treating infections caused by *S. pneumoniae* [14,24]. Furthermore, although tolerances to vancomycin were obtained based on accurate experimental studies, the determination and interpretation of vancomycin tolerance is laborious and may pose difficulties for medical diagnostic laboratories. The results of the present study have revealed that the Etest and microdilution methods for determination vancomycin MIC can be easily performed and are predictive indicators for the tolerance or decrease of susceptibility to vancomycin.

In this regards, vancomycin MIC determination for pathogenic ceftazidime resistant *S. pneumoniae* strains has not been reported up to present. The results of vancomycin MIC determination of different *S. pneumoniae* serotypes were shown in Table 2. The MIC of vancomycin for strains belonging to serotypes 2 and 4 revealed a maximum zone of inhibition of 21 and 10 millimeter in diameter, respectively. These two serotypes were isolated from the lung. The vancomycin MIC of 10 strains (20% of isolates) (serotypes 1, 2, 4, 5, 6, and 19) was equal to 0.38 μg/ml of vancomycin, with a zone of inhibition of 20–29 millimeters in diameter. The MIC of 14 different serotypes (28% of isolates) were equal to 0.5 − 0.75 μg/ml of vancomycin, with a zone of inhibition of 15–25 millimeter in diameter. The MIC for other 14 different serotypes (28% of isolates) was equal to 1 μg/ml of vancomycin, corresponding to a zone of inhibition of 10–30 millimeters in diameter. Three serotypes (6% isolates) have MICs equal to 1.5 μg/ml, with a zone of inhibition of 1–3 centimeters in diameter. Two isolates (4%) of the serotype 20 had a MIC of 4 and 6 μg/ml, while the MICs of serotypes 14 and 17 (4%) were 5 and 8 μg/ml, respectively, with a zone of inhibition equal to 10 millimeter in diameter. According to the CLSI guidelines, a MIC of 2 μg/ml of vancomycin, with a zone of inhibition ≤17 millimeters should be regarded as a resistant strain [18]. However, the sensitive ranges of a vancomycin MIC is less 2 μg/ml. It can be suggested that in this study 46 *S. pneumoniae* strains (92%) were vancomycin susceptible, while four strains (8%) manifested a decreasing susceptibility to vancomycin.

A very important finding of the current study was that the isolates presenting a decreasing susceptibility to vancomycin were also resistant to ceftazidime. In addition, the results of our previous study showed that nine *S. pneumoniae* strains (18%) were resistant to penicillin [19]. The results of this study indicate that only four out of 50 ceftazidime resistant of *S. pneumoniae* strains manifested decreased susceptibility to vancomycin.

In 2010, Moscoso et al. showed that the vancomycin tolerance in *S. pneumoniae* isolates depends on reduced enzyme activity of the major LytA autolysin or capsular polysaccharide [11]. In this study, the PCR methods were not able to determine the *van*A or *van*B gene, which induced vancomycin resistance. Despite the present study, Kohanteb and Sadeghi (2007) reported that vancomycin MIC for penicillin-resistant *S. pneumoniae* strains in Iran was 0.03 − 0.5 μg/ml [25]. Bokaeian et al. (2011) reported that the vancomycin MIC of *S. pneumoniae* from nasopharyngeal carriage among healthy adolescents in Zahedan was 0.02 − 1 μg/ml, and concluded that all *S. pneumoniae* isolates were susceptible to vancomycin [26]. However, Sanaei DA et al., which studied the nasopharyngeal carrier rate of *S. pneumoniae* in children by using the disc diffusion method, showed that 1.5% of *S. pneumonia*e isolates were non susceptible to vacomycin [27].

## Conclusion

However, have not access to positive and negative control strains was the main limitation of this investigation. the results of this study revealed that 92% of pathogenic ceftazidime resistant strains of *S. pneumoniae* have vancomycin MICs of 0.19 − 1.5 μg/ml and only 8% of them have a vancomycin MIC of 4–8 μg/ml. Based on the data collected from this research, the later serotypes had been marked as vancomycin tolerant or may be vancomycin non- susceptible strains. However, more ample genetic and molecular research are needed, as these findings represent an important warning to health authorities because the occurrence of *S. pneumoniae* strains with multiple resistances has been shown, a situation which has never been reported before.
